# The Effect of Light on Plastid Differentiation, Chlorophyll Biosynthesis, and Essential Oil Composition in Rosemary (*Rosmarinus officinalis*) Leaves and Cotyledons

**DOI:** 10.3389/fpls.2020.00196

**Published:** 2020-03-03

**Authors:** Andrea Böszörményi, Adrienn Dobi, Anna Skribanek, Melinda Pávai, Katalin Solymosi

**Affiliations:** ^1^Department of Pharmacognosy, Semmelweis University, Budapest, Hungary; ^2^Department of Plant Anatomy, ELTE Eötvös Loránd University, Budapest, Hungary; ^3^Department of Biology, ELTE Savaria University Centre, Szombathely, Hungary

**Keywords:** dark-forcing, etiolation, leucoplast, peltate glandular hair, prolamellar body, rosemary, tubular complex

## Abstract

It is unclear whether light affects the structure and activity of exogenous secretory tissues like glandular hairs. Therefore, transmission electron microscopy was first used to study plastid differentiation in glandular hairs and leaves of light-grown rosemary (*Rosmarinus officinalis* “Arp”) plants kept for 2 weeks under ambient light conditions. During our detailed analyses, among others, we found leucoplasts with tubuloreticular membrane structures resembling prolamellar bodies in stalk cell plastids of peltate glandular hairs. To study the effect of darkness on plastid differentiation, we then dark-forced adult, light-grown rosemary plants for 2 weeks and observed occasionally the development of new shoots with elongated internodes and pale leaves on them. Absorption and fluorescence spectroscopic analyses of the chlorophyllous pigment contents, the native arrangement of the pigment-protein complexes and photosynthetic activity confirmed that the first and second pairs of leaf primordia of dark-forced shoots were partially etiolated (contained low amounts of protochlorophyll/ide and residual chlorophylls, had etio-chloroplasts with prolamellar bodies and low grana, and impaired photosynthesis). Darkness did not influence plastid structure in fifth leaves or secretory tissues (except for head cells of peltate glandular hairs in which rarely tubuloreticular membranes appeared). The mesophyll cells of cotyledons of 2-week-old dark-germinated rosemary seedlings contained etioplasts with highly regular prolamellar bodies similar to those in mesophyll etio-chloroplasts of leaves and clearly differing from tubuloreticular membranes of secretory cells. Analyses of the essential oil composition obtained after solid phase microextraction and gas chromatography-mass spectroscopy showed that in addition to light, the age of the studied organ (i.e., first leaf primordia and leaf tip vs. fifth, fully developed green leaves) and the type of the organ (cotyledon vs. leaves) also strongly influenced the essential oil composition. Therefore, light conditions and developmental stage are both important factors to be considered in case of potential therapeutic, culinary or aromatic uses of rosemary leaves and their essential oils.

## Introduction

Peculiar cubic phase membrane organizations, also termed tubular complexes or tubuloreticular inclusions have been observed in several animal, human or plant cells. In the former, these are thought to represent the modification of the (rough) endoplasmic reticulum, are located inside its cisternae or in the perinuclear space [e.g., Boor et al. (2018), reviewed in Luu et al. (1989), Almsherqi et al. (2006, 2009), Deng et al. (2010)] and are rich in acidic glycoproteins. In animal and human cells, the presence of tubuloreticular structures is thought to be associated with stressful or diseased conditions, however, other works suggest that they may be involved in regeneration processes of endothelial cells in wounded tissues (Eady and Odland, 1975) or are the result of altered cholesterol homeostasis induced by viral infection (Deng et al., 2010) but such structures have been also described in the aqueous lipid-protein film of lung surfactants (Larsson and Larsson, 2014). Their formation has been associated with special protein-protein interactions, overexpression of some membrane resident proteins, alterations in lipid (Almsherqi et al., 2009) or carotenoid (Park et al., 2002) composition and membrane symmetry (Larsson and Larsson, 2014), however, their exact cellular function is not known.

The formation of such cubic phase membrane structures has been also observed in semi-autonomous organelles such as mitochondria and plastids (Almsherqi et al., 2009). In the mitochondria of amoeba cells, cubic membrane formation was induced by starvation and/or autophagy processes, where they were slowing the degradation of the organelle and thus contributed to the survival of the cell upon stress conditions (Chong et al., 2017). The prolamellar bodies present in the etioplasts or etio-chloroplasts of dark-grown plants represent another well-known group of cubic phase membranes present in nature [reviewed in Solymosi and Schoefs (2010), Solymosi and Aronsson (2013), Kowalewska et al. (2019)]. Due to their very high surface-to-volume ratio, prolamellar bodies are thought to serve as a membrane reserve that can be quickly converted into a large photosynthetic membrane network upon light-dark transition of for instance seedlings germinating in the soil (Ferroni et al., 2009; Kakuszi et al., 2017) or leaf primordia of buds after bud break (Solymosi and Böddi, 2006; Solymosi et al., 2006, 2012). It has to be outlined, that prolamellar bodies represent one subtype of tubuloreticular membrane structures with highly regular spatial organization consisting of tetrahedral units of membrane tubules arranged in strictly geometric manner [reviewed in Solymosi and Schoefs (2010), Solymosi and Aronsson (2013)].

In addition to prolamellar bodies present in chlorenchyma tissues developing under light deprivation, plastids of light-grown and light-exposed tissues can also contain such peculiar membranes. Regularly arranged vesicle clusters present in chloroplasts may also form non-linear membrane organizations (Lindquist et al., 2016), and prolamellar body-like structures have been induced by UV irradiation of fruit plastids (Kovács and Keresztes, 2002), and several cells with secretory function have been reported to contain prolamellar body-like tubuloreticular membrane structures.

Such leucoplasts or secretory plastids with prolamellar body-like structures have been observed in extrafloral nectaries of *Passiflora* (Schnepf, 1961), and in plastids of glandular hairs of several genera belonging to various families [e.g., *Cannabis* – (Hammond and Mahlberg, 1978; Kim and Mahlberg, 1997; Solymosi and Köfalvi, 2017), *Artemisia* – (Ascensao and Pais, 1982), *Chrysanthemum* – (Vermeer and Peterson, 1979), *Centrolobium* – (Matos and Paiva, 2012), *Platanthera* – (Stpiczynska et al., 2005)] including Lamiaceae [e.g., *Mentha piperita* – (Amelunxen, 1965; Turner et al., 2000, 2012), *Perilla ocymoides* – (Kashina and Danilova, 1993)]. In *M*. *piperita* such structures were observed only in the stalk cells of the peltate glandular hairs (Amelunxen, 1965; Turner et al., 2000, 2012) and were observed as tubular-vesicular structures obtained only after osmium-tetroxide fixation (Amelunxen, 1965) or resembled rather plastoglobuli clusters both in conventionally chemically fixed and cryofixed material (Turner et al., 2000, 2012). In case of *Nepeta* species prolamellar body-like structures were described for the stalk cells of the peltate glandular hairs but were not shown on micrographs (Kolalite, 1998). In glandular hairs of *P*. *ocymoides* prolamellar body-like structures were reported when plants were transferred from continuous light to short-day illumination conditions, and were thus associated with processes occurring during the dark phase of growth (Kashina and Danilova, 1993). On the other hand, no cubic phase membrane structures were observed in *Lavandula* glandular hairs both when grown in light or in prolonged darkness (20 days) (Huang et al., 2008).

Our aim was to check whether such tubuloreticular structures were characteristic for the plastids of the peltate glandular hairs of other medicinally and economically important Lamiaceae species such as for example rosemary (*Rosmarinus officinalis*) (Borges et al., 2018; De Oliveira et al., 2019) and whether their presence or structure was influenced by darkness. This question is evenmore important because data about the structural and developmental plasticity of secretory plastids are rather scarce (Solymosi and Keresztes, 2012) and the role of light in the biosynthesis of the essential oil components also needs to be elucidated. This latter may have industrial or practical relevance, because during winter in colder climates rosemary plants are often brought to warmer rooms with less light but higher temperatures, while both the leaves (*Rosmarini folium*) and the essential oils (*Rosmarini aetheroleum*) of rosemary are widely used as drugs or spices.

## Materials and Methods

### Plant Material and Growth Conditions

Light-grown rosemary (*R. officinalis “*Arp”) plants with 20–40-cm-long shoots were purchased from the local market. Some plants were placed in complete darkness for 2 weeks at room temperature (20°C) – a procedure to which we will refer later to as dark-forcing because it involves adult light-grown plants which were later placed into darkness for longer periods. Other plants were kept under ambient light conditions at room temperature. Plants were watered with tap water every 4–5 days. Those being in the dark were watered using dim green safelight which was previously tested and did not induce photosynthesis or chlorophyll biosynthesis in plants. Plants purchased at the end of winter and also after the end of the long summer drought period and transferred to the dark often developed relatively large new shoots the leaves of which were used for further analyses. Rosemary has decussate leaf arrangement with a leaf pair developing at each node, but for simplicity of description later we will only refer to these pairs of leaf primordia of same developmental age and found on the same node as “first leaves” (for the youngermost pair of leaf primordia located closest to the shoot tip, on the first node) or “fifth leaves” (in case of the leaf pair located on the fifth node downward from the shoot tip), etc. It should be noted, however, that dark-forcing of rosemary plants was in other cases often unsuccessful and did not yield new shoots.

For comparison, rosemary fruits (i.e., four-nutlets) were also purchased from commercial sources (Gála, Romero alecrim, and Kerting Réde). The fruits were germinated at room temperature on wet cotton in closed nylon bags for 2 weeks either under ambient light conditions or in complete darkness. Cotyledons of such seedlings were later used for further analyses.

### Fluorescence Spectroscopy at 77 K

Youngermost and older leaves of light-grown as well as dark-forced shoots, and cotyledons of dark-grown or light-grown seedlings were separately sampled and cooled to 77 K in liquid nitrogen. To test the photoactivity of the protochlorophyll/ide pigments in the samples, the studied organs were first cooled to 77 K, measured, and then warmed up to approx. 253 K, illuminated with white light of 20 μmol s^–1^ m^–2^ photon flux density and cooled to liquid nitrogen 10 s after illumination. Low-temperature (77 K) fluorescence emission spectra were recorded using a Fluoromax-3 spectrofluorometer (Jobin Yvon-Horiba, France) with 440 nm excitation wavelength, 0.2 s integration time, 2 and 5 nm excitation and emission slits, respectively. Three spectra were recorded and automatically averaged for each sample. Emission signals were corrected for the wavelength-dependent sensitivity of the detection; when necessary, baseline correction (to eliminate light scattering effects) and 3-point and 5-point linear smoothing were also performed using SPSERV V3.14 program (copyright: C. Bagyinka, Institute of Biophysics, Biological Research Center of the Hungarian Academy of Science, Hungary). Samples were collected from at least 3 different plants, in different independent biological replicates, and representative spectra were chosen for presentation.

### Determination of Chlorophyllous Pigment Contents

The fresh mass of selected leaves or cotyledons were measured. Plant organs were then homogenized in mortar with pistil in 80% (v/v) acetone. Pigment contents of the acetonic extracts were measured using absorption spectroscopy or in some cases (e.g., samples with low pigment concentrations or simultaneous presence of protochlorophyll/ide and chlorophyllous pigments) with fluorescence spectroscopy using calibration curves. Measurements of fresh mass and pigment extraction of dark-grown or dark-forced samples were carried out under dim green safelight. The absorption of acetonic pigment extracts was determined using a UV-2101 PC (Shimadzu Corp., Japan) spectrophotometer. Spectra were recorded between 400 and 800 nm, with 0.5 nm sampling interval, 1 nm optical slits and medium speed. Spectra were used for calculation of chlorophyll a and b contents using standard equations (Porra et al., 1989). Protochlorophyll/ide quantification using calibration curve for fluorescence measurements was based on extinction coefficients as in Brouers and Michel-Wolwertz (1983), and calibration was prepared using acetonic extracts of protochlorophyll obtained from hull-less pumpkin (*Cucurbita pepo* subsp. *pepo* var. *styriaca*).

### Measurement of Photosynthetic Activity

Photosynthetic activity of various leaves of light-grown and dark-forced shoots was studied by measuring variable chlorophyll fluorescence. Due to the size of the leaves and also in order to characterize eventual spatial heterogeneity in the parameters, fluorescence images were taken with an Imaging PAM-M series Chlorophyll Fluorometer (Heinz Walz GmbH, Germany). Dark-forced samples were positioned into the instrument and the camera focus was set under dim green safelight. Prior to the measurements light-grown samples were dark adapted for 15 min. The photosynthetic parameter F_*v*_/F_*m*_ = (F_*m*_-F_0_)/F_*m*_ (Björkman and Demmig, 1987) was calculated in at least four biological replicates as in Solymosi et al. (2007). Average values and standard deviations are provided. It is important to note that these parameters are based on optical properties of green leaves and are probably different in dark-forced tissues with lower chlorophyll content, therefore, these values should be regarded only as an estimation.

### Transmission Electron Microscopy (TEM)

Leaves were cut into 1 mm × 2 mm or 1 mm × 1 mm pieces at the central leaf blade region (i.e., avoiding leaf base and leaf tip regions, but also the midrib) with sharp razor blade in drops of the primary fixative, 2.5% glutaraldehyde in 70 mM Na_2_HPO_4_-KH_2_PO_4_ buffer (pH = 7.2). Intact cotyledons were simply separated from the hypocotyl and put in the same primary fixative. After 3 h of fixation in glutaraldehyde, pieces of the plant material were rinsed three times for 15 min in the same phosphate buffer, and were then post-fixed for 1 h in 1% osmium tetroxide dissolved in the same buffer. After rinsing with the phosphate buffer for 15 min three times, samples were dehydrated in ethanol series, transferred to propylene-oxide and embedded in Durcupan ACM resin (Fluka Chemie AG, Switzerland). Ultrathin sections (70 nm) were prepared on a Reichert Jung Ultracut-E ultramicrotome (Reichert Jung AG, Austria) and were stained with uranyl acetate and Reynold’s lead citrate. Transmission electron microscopic (TEM) analyses were carried out with a Hitachi 7100 TEM (Hitachi, Japan) at 75 kV accelerating voltage and a JEOL JEM 1011 (JEOL Ltd., Japan) at 80 kV accelerating voltage. Digital images were taken using Olympus Morada CCD cameras (Olympus Optical Co. Ltd., Japan). Fast Fourier transformation (FFT) on the selected region of interest of particular micrographs was performed using ImageJ software.

### Solid Phase Microextraction (SPME) of the Essential Oil

Plant material (pooled samples of 0.5–1 g fresh mass of either several fully developed, i.e., mature leaves, or young leaves and shoot tips or cotyledons as indicated) was put into vials (20 mL headspace) sealed with a silicon/polytetrafluoroethylene septum prior to the SPME gas chromatography/mass spectrometry (SPME-GC/MS) analysis. Sample preparation using the static headspace solid phase microextraction (sHS-SPME) technique was carried out with a CTC Combi PAL (CTC Analytics AG, Switzerland) automatic multipurpose sampler using a 65 μM StableFlex polydimethyl siloxane/carboxene/divinyl benzene (CAR/PDMS/DVB) SPME fiber (Supelco, United States). After an incubation period of 5 min at 100°C, extraction was performed by exposing the fiber to the headspace of a 20 mL vial containing the sample for 10 min at 100°C. The fiber was then immediately transferred to the injector port of the GC/MS, and desorbed for 1 min at 250°C. Injections were made in splitless mode. The SPME fiber was cleaned and conditioned in a Fiber Bakeout Station in pure nitrogen atmosphere at 250°C for 15 min.

### Gas Chromatography-Mass Spectroscopy (GC/MS) Conditions

The analyses were carried out with an Agilent 6890N/5973N GC/MSD (Agilent, United States) system equipped with an Supelco (Sigma-Aldrich) SLB-5MS capillary column (30 m × 250 μm × 0.25 μm). The GC oven temperature was programmed to increase from 60°C (3 min isothermal) to 250°C at 8°C/min (1 min isothermal). High purity helium (6.0) was used as carrier gas at 1.0 mL/min (37 cm/s) in constant flow mode.

The mass selective detector (MSD) was equipped with a quadrupole mass analyzer and was operated in electron ionization mode at 70 eV in full scan mode (41–500 amu at 3.2 scan/s).

The data were evaluated using MSD ChemStation D.02.00.275 software (Agilent). The identification of the compounds was carried out by comparing retention data and recorded spectra with literature data, and the NIST 2.0 library was also consulted. The percentage evaluation was carried out by area normalization. Averaged data and their standard errors are from at least 3 independent biological replicates as indicated.

### Statistical Analyses

The data in Tables 1, 2 represent the mean ± the standard error of the mean of the number of independent biological replicates obtained on pooled samples from different experiments as indicated. The datasets obtained in different samples and treatments were compared using the software GraphPad InStat (GraphPad Software Inc., United States) using 1-way ANOVA followed by Tukey-Kramer multiple comparisons test as posterior test where significant differences were found. Significant differences are described in the text and designated with different letters in the Tables 1, 2 at *P* < 0.05.

**TABLE 1:** Pigment contents of different organs of light-grown adult rosemary7.7pc plants grown for 2 weeks in complete darkness (“dark-forced”) or under ambient light conditions (“light-grown”).

	**Light-grown (*n* = 3–6)**			**Dark-forced (*n* = 5–6)**		
	**Chlorophyll a (μg/g)**	**Chlorophyll b (μg/g)**	**Chlorophyll a/b**	**Chlorophyll a (μg/g)**	**Chlorophyll b (μg/g)**	**Chlorophyll a/b**
Shoot tip and first leaf primordia	663.85 ± 95.62^a^	203.70 ± 33.79^a^	3.35 ± 0.12^a^	66.42 ± 14.47^b^	21.38 ± 4.32^b^	3.08 ± 0.11^ab^
Second leaf primordia	700.60 ± 10.72^a^	207.36 ± 6.70^a^	3.38 ± 0.09^a^	286.14 ± 19.47^c^	82.63 ± 6.5^c^	3.47 ± 0.06^a^
Fifth leaf	728.09 ± 88.87^a^	243.25 ± 29.49^a^	2.99 ± 0.04^b^	623.14 ± 46.19^a^	211.48 ± 13.70^a^	2.95 ± 0.07^b^

*Average pigment contents and their standard errors are provided on a fresh mass basis for pooled samples and the number of parallels as indicated. Data were calculated from absorption measurements of 80% acetonic extracts of the leaves. In case of dark-forced plants the newly developed pale shoots were studied. Different superscript letters indicate significant difference in the chlorophyll a or chlorophyll b or chlorophyll a/b ratio of the different organs (shoot tip and leaf primordia, second leaf primordia and fifth leaf) and treatments (light-grown or dark-forced) according to 1-way-ANOVA followed by Tukey-Kramer multiple comparison test (*P* < 0.05).*

**TABLE 2:** Percentage composition of the essential oils produced by the cotyledons of dark-germinated or light-germinated 2-week-old rosemary seedlings and of different leaves of adult rosemary plants kept for 2 weeks under ambient light conditions (“light-grown” plants) or in complete darkness (“dark-forced”) at room temperature.

**Compounds**	**Retention indices**	**Percentage ratio of the compounds (%)**					
		**Leaves of adult plants**				**Cotyledons of seedlings**	
		**Dark-forced, young (*n* = 4)**	**Dark-forced, old (*n* = 3)**	**Light-grown young (*n* = 3)**	**Light-grown, old (*n* = 4)**	**Dark-germinated (*n* = 3)**	**Light-germinated (*n* = 3)**
α-Pinene	939	4.8 ± 0.6^a^	23.2 ± 3.0^b^	9.8 ± 2.7^c^	25.2 ± 2.1^b^	3.0 ± 0.9^a^	3.8 ± 0.6^a^
Camphene	954	3.1 ± 0.3^a^	4.6 ± 0.7^bc^	4.8 ± 0.7^bc^	5.1 ± 0.6^b^	3.0 ± 0.8^a^	3.7 ± 0.4^ac^
Sabinene	975	0.1 ± 0.0^a^	0.8 ± 0.2^b^	0.2 ± 0.1^a^	1.0 ± 0.2^b^	–	–
β-Pinene	979	4.3 ± 0.5^a^	1.4 ± 0.8^b^	6.0 ± 1.0^ac^	1.2 ± 0.6^b^	12.2 ± 2.6^d^	8.3 ± 0.2^c^
3-Octanone	984	0.1 ± 0.0^a^	1.4 ± 0.5^b^	0.1 ± 0.1^a^	2.1 ± 0.6^b^	–	–
Myrcene	991	1.0 ± 0.1^a^	3.6 ± 0.4^b^	1.5 ± 1.0^a^	3.7 ± 0.3^b^	–	–
α-Terpinene	1017	0.1 ± 0.0^a^	0.7 ± 0.2^b^	0.5 ± 0.1^b^	0.6 ± 0.1^b^	–	–
p-Cymene	1025	0.1 ± 0.0^a^	1.0 ± 0.2^b^	0.2 ± 0.1^a^	1.1 ± 0.3^b^	–	–
Limonene	1029	1.7 ± 0.2^a^	4.6 ± 0.3^b^	2.3 ± 0.5^a^	4.7 ± 0.3^b^	0.4 ± 0.2^a^	1.3 ± 0.1^a^
1,8-Cineole	1031	4.2 ± 0.1^a^	8.3 ± 0.8^b^	7.0 ± 0.6^b^	7.8 ± 1.0^b^	3.7 ± 0.2^a^	2.8 ± 0.2^a^
γ-Terpinene	1060	0.6 ± 0.1^a^	1.1 ± 0.4^a^	0.9 ± 0.2^a^	0.9 ± 0.4^a^	–	0.2 ± 0.1^b^
Terpinolene	1089	0.7 ± 0.1^a^	1.4 ± 0.4^b^	0.9 ± 0.1^a^	1.3 ± 0.5^b^	–	–
Linalool	1097	0.3 ± 0.4^a^	1.8 ± 0.2^b^	0.2 ± 0.1^a^	1.2 ± 0.3^b^	–	—
Chrysanthenone	1128	0.1 ± 0.0^a^	1.7 ± 1.2^b^	0.2 ± 0.1^a^	0.9 ± 0.4^ab^	–	–
Camphor	1146	4.0 ± 0.5^a^	7.3 ± 1.0^b^	5.9 ± 0.8^b^	6.7 ± 1.1^b^	2.6 ± 0.6^a^	3.8 ± 0.2^a^
Pinocamphon	1163	0.6 ± 0.1^a^	0.5 ± 0.4^a^	0.4 ± 0.2^a^	0.5 ± 0.2^a^	–	–
Borneol	1169	4.2 ± 0.9^a^	4.3 ± 0.4^a^	3.1 ± 1.6^a^	3.6 ± 0.7^a^	–	0.3 ± 0.2^b^
Isopinocamphon	1175	2.0 ± 0.7^a^	1.1 ± 0.1^b^	2.2 ± 0.2^a^	1.0 ± 0.1^b^	–	–
Terpinen-4-ol	1177	0.9 ± 0.1^a^	1.1 ± 0.1^a^	0.9 ± 0.2^a^	0.9 ± 0.1^a^	–	–
α-Terpineol	1189	0.6 ± 0.1^a^	1.5 ± 0.2^b^	0.7 ± 0.2^a^	1.3 ± 0.1^b^	–	–
Dihydrocarvone	1201	0.5 ± 0.1^a^	0.6 ± 0.1^a^	0.5 ± 0.1^a^	0.5 ± 0.1^a^	–	–
Verbenone	1205	1.4 ± 0.3^a^	8.4 ± 1.9^b^	2.5 ± 1.7^a^	8.2 ± 1.4^b^	–	–
Carvone	1243	0.7 ± 0.1^a^	1.0 ± 0.3^a^	0.7 ± 0.3^a^	0.8 ± 0.3^a^	–	–
Bornyl acetate	1289	26.1 ± 0.8^a^	2.1 ± 1.2^b^	14.6 ± 4.9^a^	2.5 ± 2.1^b^	43.8 ± 5.6^c^	37.0 ± 9.6^c^
Ylangene	1375	–	–	–	–	1.2 ± 1.0^a^	2.4 ± 0.5^a^
β-Caryophyllene	1409	22.1 ± 1.3^a^	13.3 ± 2.6^b^	22.3 ± 1.8^a^	13.1 ± 2.7^b^	3.2 ± 0.1^c^	4.3 ± 0.9^c^
Aristolene	1416	–	–	–	–	2.8 ± 0.9^a^	2.3 ± 0.5^a^
α-Humulene	1455	3.7 ± 0.1^a^	1.9 ± 0.3^bc^	2.7 ± 1.0^ac^	1.9 ± 0.3^bc^	0.2 ± 0.1^d^	1.0 ± 0.4^*cd*^
γ-Muurolene	1480	–	–	–	–	3.1 ± 1.2^a^	3.2 ± 2.2^a^
β-Muurolene	1493	–	–	–	–	1.5 ± 1.3^a^	2.1 ± 1.3^a^
δ-Cadinene	1529	–	–	–	–	8.6 ± 2.2^a^	11.5 ± 1.0^a^
Caryophyllene oxide	1583	7.0 ± 0.5^a^	0.8 ± 0.3^b^	4.2 ± 1.1^c^	0.7 ± 0.3^b^	0.7 ± 0.2^b^	1.4 ± 1.3^b^
τ-Cadinol	1640	–	–	–	–	5.7 ± 4.1^a^	4.5 ± 2.9^a^
Monoterpenes		61.8	83.2	66.2	82.5	68.7	61.3
Sesquiterpenes		32.7	16.0	29.2	15.7	27.0	32.7
Hydrocarbons		42.1	57.4	52.1	59.8	39.1	44.2
Oxigen containing compounds		52.4	41.8	43.3	38.4	56.5	49.8
Alcohols		5.9	8.7	4.9	7.0	5.7	4.9
Ketons		9.2	22.0	12.6	20.5	2.6	3.8
Peroxides		7.0	0.8	4.2	0.7	0.7	1.4
Ethers		4.2	8.3	7.0	7.8	3.7	2.8
Esters		26.1	2.1	14.6	2.5	43.8	37.0

*From adult plants fully developed fifth leaves (“old”) as well as first leaf primordia (“young”) were also analyzed. In case of dark-forced plants only the leaves and shoot tips of newly formed, pale shoots were studied. The experiments were carried out in three or four independent biological replicates as indicated, and for each measurement pooled samples (0.5–1 g plant material) were used. Standard errors of the mean are indicated in the Table. Different letters in each row indicate significant difference according to 1-way-ANOVA followed by Tukey-Kramer multiple comparison test (*P* < 0.05).*

## Results and Discussion

### Ultrastructural Analyses of the Glandular Hairs of Light-Grown Rosemary Leaves

**FIGURE 1:**
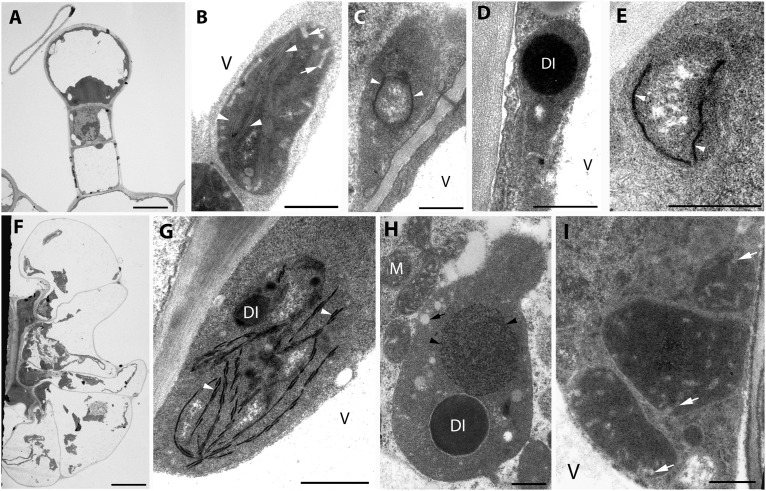
Plastid ultrastructure in capitate **(A)** and peltate **(F)** glandular hairs of light-grown rosemary (*Rosmarinus officinalis*). Representative plastids from the basal (epidermal) cell **(B)**, stalk cells (lower cell: **C**, upper cell: **D**), head cell **(E)** of capitate glandular hairs. Plastids in the basal **(G)**, stalk **(H)**, and disc (i.e., head) cells **(I)** of peltate glandular hairs. Scale bar: 5 μm **(A,F)**, 0.5 μm **(B–E,G–I)**. DI, dense plastid inclusion; M, mitochondrium; V, vacuole; black arrow, plastoglobule-like electron-transparent inclusion; black arrowhead, tubuloreticular membrane; white arrow, invagination of the envelope; white arrowhead, thylakoid membrane.

Fully differentiated capitate (Figure 1A) and peltate (Figure 1F) glandular hairs were present and studied both on mature and on young green leaves as reported earlier (Werker et al., 1985; Marin et al., 2006; Boix et al., 2011). Below we describe plastid ultrastructure in these secretory structures in rosemary. Plastid stroma was in general very electron-dense, and the contrast between the cytoplasm and the plastid stroma was often low. Plastids of basal cells of capitate glandular hairs resembled typical epidermal chloroplasts, they contained few stacked thylakoids, i.e., grana with so-called “inverse contrast” corresponding in fact to lightly staining membranes containing dense luminal substance (Keresztes and Sárvári, 2001), and several plastid invaginations indicating active material transport with the cytoplasm (Figure 1B). Stalk and head cell plastids were leucoplasts and had similar structure (Figures 1C–E) in capitate glandular hairs: they contained only few single thylakoid-like structures, and sometimes electron-dense dark inclusions.

The basal cells of the fully developed peltate glandular hairs had chloroplasts with electron-dense thylakoids, low grana and sometimes dark inclusions (Figure 1G). The plastids in the stalk cell contained in general one large electron-dense inclusions, and electrontransparent ovale bodies resembling plastoglobuli (Figure 1H). Often electron-dense tubuloreticular membranes in oval structures with average diameter of approx. 1.2 μm were also present (Figure 1H). Plastids of the disc cells were leucoplasts with electron-dense stroma and several invaginations during the secretory phase (Figure 1I).

The observed plastid morphological features (dense inclusions, electron-dense stroma, etc., summarized in Supplementary Table S1) are consistent with literature data about the plastids of capitate trichomes [*Lavandula*– (Huang et al., 2008), *Leonotis* – (Ascensao and Pais, 1998), *M*. *piperita* – (Amelunxen, 1965), *Nepeta* – (Kolalite, 1998), *Stachys* – (Giuliani et al., 2008)] and peltate trichomes [*Lavandula* – (Huang et al., 2008), *Leonotis* – (Ascensao et al., 1997), *M*. *piperita* – (Amelunxen, 1965; Turner et al., 2000, 2012), and *Nepeta* – (Kolalite, 1998)] of other light-grown Lamiaceae plants. It should be noted, however, that in case of stalk cells, the observed tubuloreticular membrane structure is different from that previously reported for *Mentha* (Amelunxen, 1965; Turner et al., 2000, 2012), in rosemary this structure is more clearly related to tubular complexes and bears no resemblance to clusters of plastoglobuli. The observed electron-dense inclusions present in plastids may be of any osmiophilic material (e.g., phenolic or terpenoid substances), however, the fact that their surface boundary is not completely smooth and that in our case (especially in stalk cells of peltate trichomes) they seem to be surrounded by a less electron-dense layer make it unlikely that they are “large plastoglobuli” (Turner et al., 2000) or “lipid droplets” in plastids (Huang et al., 2008).

The factors influencing the formation of these tubuloreticular membranes in stalks cells remain unknown. The basal cells of the trichomes (Figure 1G) and the epidermis cells located directly below the peltate glandular hairs (not shown) contained chloroplasts, which indicates that these cells were exposed to light and could thus produce chloroplasts. In this context, it is unlikely that only the stalk cells of the peltate glandular hairs were somewhat partially or fully shaded by the head cells and their secreted material, resulting in their etiolation and consequent formation of tubuloreticular membranes. Similarly, we had no information about the structure of “real” prolamellar bodies of rosemary, therefore, we decided to check how complete darkness influenced plastid differentiation in rosemary leaves.

### The Effect of Prolonged Darkness (i.e., Dark-Forcing and Etiolation) on Plastid Differentiation in Rosemary Leaves

There are literature data describing plastid developmental differences and prolamellar body formation along decreasing light gradients being present in different cells of partly shaded leaf primordia in buds (Solymosi et al., 2012) or in sunflower cotyledons covered by the pericarp (Solymosi et al., 2007). Therefore, it may be possible that the disc cells and their secretory products may shade the stalk cell plastids and thus influence their differentiation. Literature data about glandular hair development and plastid structure under prolonged darkness are scarce and somewhat conflicting. In case of *P*. *ocymoides* the presence of tubuloreticular inclusions was suggested to be associated with cultivation under short-day conditions and thus increased dark periods (Kashina and Danilova, 1993), while in case of *Lavandula pinnata* no tubuloreticular structures have been observed in axillary bud-derived explants cultivated for 20 days *in vitro* on Murashige-Skoog medium both in the light (low light, i.e., 10–20 μmol photons s^–1^ m^–2^) or in complete darkness for 20 days (Huang et al., 2008) and light did not influence glandular hair development and structure (Huang et al., 2008). Therefore, the question can be raised, whether the formation of the tubuloreticular membranes in the plastids of the stalk cells of peltate glandular hairs and its absence in other secretory plastids is influenced by light or not.

**FIGURE 2:**
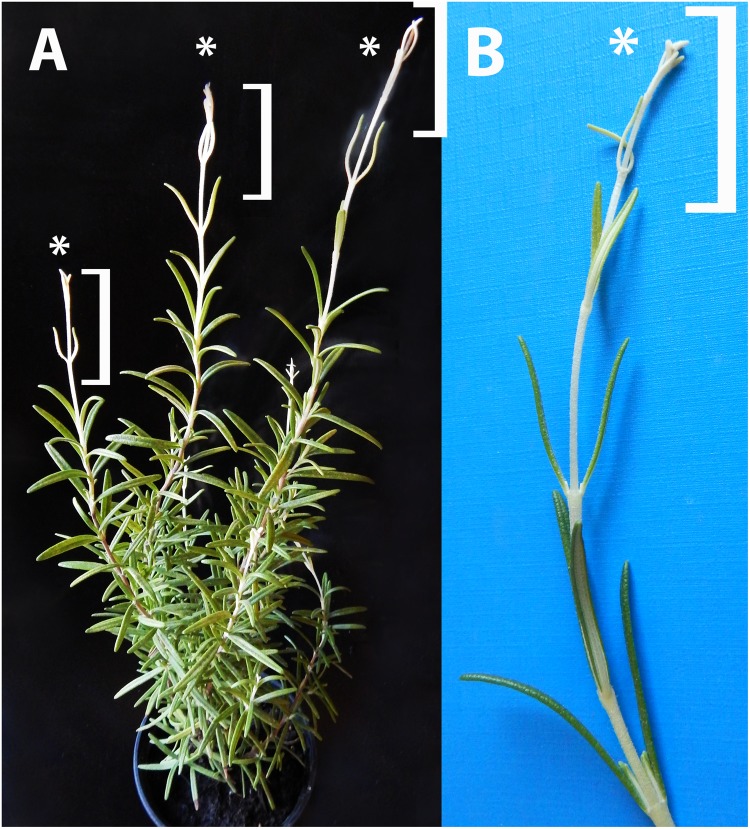
Dark-forced rosemary (*R. officinalis*) plant **(A)** with new, dark-grown shoots (asterisks and square brackets) which developed during 2 weeks of growth in complete darkness. These new shoots **(B)** have pale, whitish-yellowish etiolated leaves and elongated stems which developed from the shoot apical meristems on the light-grown plant.

In order to check this, light-grown rosemary plants were transferred to full darkness for 2 weeks. From the shoot apical meristems or axillary buds of some plants, dark-forced shoots developed which had long internodes and smaller, pale green or whitish-yellowish leaves (Figure 2A). The original, oldest light-grown leaves had dark green color, followed by leaves with pale green color which were only partially dark-forced, and the youngest leaves close to the leaf tip were whitish-yellowish on both sides (Figure 2B).

**FIGURE 3:**
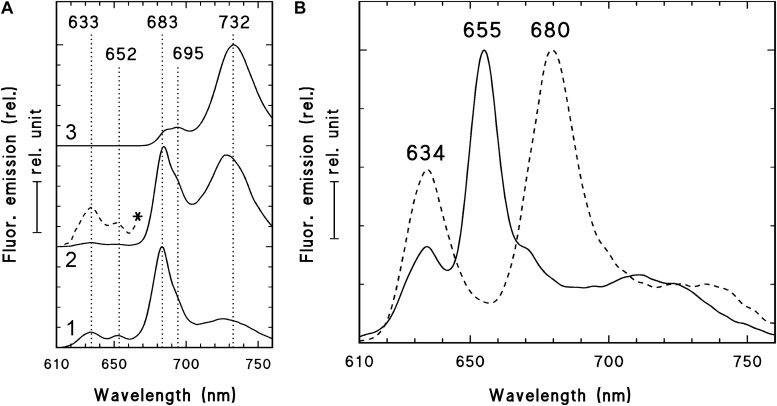
77 K fluorescence emission spectra of the shoot tip and first leaf primordia (1), second leaf primordia (2), and third leaves (3) of 2-week-old dark-forced shoots of rosemary plants (as shown close to the asterisk in Figure 2B) **(A)** and 2-week-old dark-germinated cotyledons **(B)**. After measurement at 77K, the dark-germinated cotyledon (**B**, solid line) was warmed to 253 K in dim green safelight, illuminated for 10 s with white light of 20 μmol s^–1^ m^–2^ photon flux density, cooled to 77 K after 10 s in the dark and measured again (**B**, broken line). The spectra were normalized at their maxima and in case of the panel **(A)** they were shifted along the *y* axis for better presentation. In case of spectrum 2 **(A)** the spectrum region between 610 and 670 nm was multiplied by 10 and is also shown (*, broken line). Excitation wavelength: 440 nm.

In order to check and confirm that these leaves developed in full darkness for 2 weeks, the shoot tip and different leaf primordia of dark-forced plants (Figure 2B, the leaves at the level of the asterisk or below) were collected in dim green safelight and cooled to 77 K in liquid nitrogen. The fluorescence emission spectra of the shoot tip and first leaves were recorded using 440 nm excitation and have shown major fluorescence emission bands at 633, 652 and 683 nm (Figure 3A, curve 1). When compared to the first leaves, the relative contribution of the short wavelength fluorescence bands at 633 and 652 nm decreased in the second leaf primordia, and a new band appeared at 730 nm (Figure 3A, curve 2). The third leaf primordia (Figure 3A, curve 3) only had chlorophyll fluorescence emission bands at 685, 695 and 732 nm, which are characteristic to leaves with fully developed and functional photosynthetic apparatus, and correspond to the fluorescence of the major chlorophyll-protein complexes of PSII core antenna (685 and 695 nm) and PSI light-harvesting complex (735) (Briantais et al., 1986). Similar spectra typical for green leaves were recorded in older leaves (i.e., third leaves and fully developed, fifth leaves and below) of the dark-forced shoots as well as in the shoot tip and all different leaves of light-grown plants, and in light-germinated cotyledons (not shown).

For comparison, the fluorescence emission spectra of the cotyledons of dark-germinated seedlings were also recorded (Figure 3B, solid line). In these spectra fluorescence bands were only observed at 634 and 655 nm and no chlorophyll fluorescence emission bands were observed, indicating that these tissues developed in complete darkness and were thus fully etiolated. Upon warming up and illumination and subsequent repeated freezing of the cotyledons, the band at 655 nm disappeared and a new band appeared at around 680 nm, indicating the transformation of photoactive protochlorophyllide pigments into chlorophyllide. Similar transformation (i.e., disappearance) of the fluorescence emission band at 652 nm was observed in the first and second leaves of dark-forced shoots upon illumination (not shown).

The fluorescence spectra of dark-forced first and second leaf primordia and the shoot tip had a chlorophyll band at 683 nm with a small shoulder at 695 nm (seen as the assymetry of the band at 683 nm), and the second leaf primordia also had a fluorescence band at around 730 nm. This indicates that these tissues were not completely devoid of chlorophylls, but probably contained remnants of chlorophylls that were present in the plastids of the light-exposed shoot apical meristem before dark-forcing and have been carried over by plastid divisions to the dark-forced tissues similarly to earlier observations in other species (Böddi et al., 1999; Solymosi et al., 2004, 2006). In addition, the dark-forced first and second leaf primordia evidently showed the presence of protochlorophyll/ide fluorescence emission at 652 and 633 nm, which originates from photoactive and non-photoactive protochlorophyll/ide spectral forms, i.e., protochlorophyllide bound to NADPH:protochlorophyllide oxidoreductase (POR) or to other proteins, respectively (Böddi et al., 1989; Solymosi and Schoefs, 2010; Solymosi and Aronsson, 2013). The accumulation of these pigments, especially that of the photoactive protochlorophyllide form with emission maximum at around 652 nm that is converted to chlorophyllide upon μs time scale of illumination and thus disappears when exposed to light (Scrutton et al., 2012), proved that during dark-forcing, the previously light-grown tissues accumulated considerable amounts of a chlorophyll precursor, and had inhibited chlorophyll biosynthesis due to the lack of light (Solymosi and Schoefs, 2010; Solymosi and Aronsson, 2013). The simultaneous presence of protochlorophyll/ide and chlorophyll pigments is typical for tissues that have been illuminated earlier during their development and were dark-forced or etiolated later only transiently (Solymosi et al., 2006, 2007, 2012; Schoefs and Franck, 2008) or completely (Böddi et al., 1999; Solymosi et al., 2004). The accumulation of photoactive protochlorophyllide confirms that these dark-forced rosemary plants developed in the absence of light during 2 weeks. The 77K fluorescence emission spectra showed clearly that dark-germinated cotyledons were fully etiolated and did not receive any light during their germination, while the leaf primordia which developed on previously illuminated (light-grown) shoot apex developed in complete darkness and got thus dark-forced for 2 weeks, but still contained chlorophyll-protein complexes that were probably transmitted to them via plastid division.

In order to quantify the pigment contents of the various studied tissues, acetonic pigment extraction was performed and the pigment contents were determined using absorption spectroscopy (Table 1). Furthermore, fluorescence spectroscopic measurements of the acetonic extracts enabled the detection of approx. 1 μg/g protochlorophyll/ide in the leaf tip and first leaf primordia of dark-forced shoots. The protochlorophyll/ide content of the second leaf primordia was beyond the detection limit of the instrument. For comparison, the fully etiolated cotyledons of 2-week-old seedlings contained 7.87 ± 0.78 μg/g protochlorophyll/ide (*n* = 7), and the light-grown cotyledons of 2-week-old seedlings contained 942.57 ± 42.69 μg/g chlorophyll a, 308.22 ± 22.23 μg/g chlorophyll b, and had a chlorophyll a/b ratio around 3.11 ± 0.12 (*n* = 8).

In case of light-grown shoots only a very slight (and not significant) increase in pigment contents was observed from the shoot tip and first leaf primordia toward the fully green fifth leaves, indicating that chlorophyll biosynthesis almost fully proceeded during 2 weeks in young leaf primordia. On the other hand, the pigment contents of the dark-forced shoot tip and first leaf primordia, and the second leaves were one tenth and one third of the corresponding light-grown organs, while that of the fifth leaves was only slightly, and not significantly lower in the dark than in the light. This indicates that chlorophyll biosynthesis was inhibited in the dark-forced young tissues, and the chlorophyll present in these tissues originates probably from the originally light-exposed shoot tip. In these dark-forced tissues protochlorophyll/ide accumulated to moderate levels similarly to partially or transiently etiolated or naturally dark-forced or etiolated tissues (Solymosi et al., 2004, 2006, 2007, 2012; Solymosi and Böddi, 2006; Schoefs and Franck, 2008). The relatively low chlorophyll contents of rosemary observed in this work when compared to chlorophyll contents of light-grown organs of other plants (Solymosi et al., 2004, 2006, 2007, 2012; Solymosi and Böddi, 2006; Schoefs and Franck, 2008) may be related to the presence of phenolic or terpenoid compounds which may interfere with acetonic pigment extraction (Dumbravǎ and Moldovan, 2012). Photosynthetic pigment extraction from such plant material is challenging (Dumbravǎ and Moldovan, 2012) and may thus require further methodological development and optimalization. However, using the available standard pigment extraction protocol (Porra et al., 1989), our data can be compared across treatments. The pigment content analyses confirmed that chlorophyll biosynthesis proceeded in the light-grown shoots (e.g., also in the shoot tip and first leaf primordia), while the same process was partially inhibited in the shoot tip and first and second leaf primordia of dark-forced shoots during 2 weeks of dark development.

Photosynthetic activity was also compared in the different leaves of dark-forced and light-grown shoots. The maximum quantum yield of PSII (F_*v*_/F_*m*_) was significantly lower in the dark-forced shoots and first leaves (0.61 ± 0.04), while in second (0.74 ± 0.03) and fifth leaves (0.76 ± 0.01) of dark-forced shoots it was similar to values found in first (0.79 ± 0.01) and fifth leaves (0.77 ± 0.01) of light-grown shoots, indicating impaired photosynthetic activity in the young dark-forced leaves when compared to light-grown leaves. These data show that the significantly lower chlorophyll content (Table 1) and different native organization of the photosynthetic pigments (Figure 3A) was also accompanied by significantly lower quantum efficiency of the photosynthetic apparatus only in the youngermost leaves and the shoot tip of the dark-forced shoots. However, although the more developed photosynthetic apparatus remained stable during 2 weeks of dark-forcing in older leaves, due to the absence of light photosynthesis was inhibited and thus the plants had to live heterotrophically using their food reserves. The question could be raised how this influenced plastid structure in these tissues.

**FIGURE 4:**
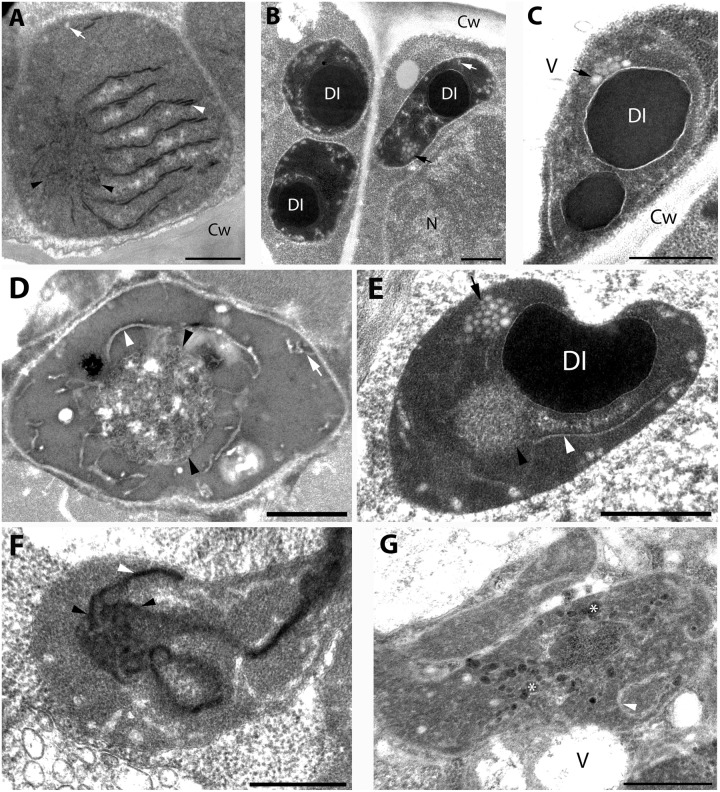
Plastid ultrastructure in first leaf primordia of dark-forced rosemary (*R. officinalis*) shoots, i.e., newly developed shoots formed on adult plants during 2 weeks of growth in complete darkness. Plastids are from leaf mesophyll cell **(A)**, epidermis cell **(B)**, and peltate glandular hairs **(C–F)**. Plastid(s) from basal cell **(C)**, stalk cells **(D,E)**, head cells **(F,G)**. Scale bar: 0.5 μm. DI, dense plastid inclusion; Cw, cell wall; N, nucleus; V, vacuole; asterisk, electron-dense membrane bound inclusion; black arrow, plastoglobule-like electron-transparent inclusion; black arrowhead, tubuloreticular prolamellar body-like membrane; white arrow, peripheral vesicle or invagination of the envelope; white arrowhead, thylakoid membrane.

Analyses of plastid ultrastructure in the first leaf primordia of these dark-forced shoots have shown that leaf mesophyll cells contained plastids with dark, electron-dense stroma, not highly regular or clearly distinguishable prolamellar bodies (average diameter: 0.7 μm), few peripheral vesicles or invaginations and thylakoids with “inverse contrast” that were sometimes stacked (mostly as bithylakoids or low grana consisting of three thylakoid layers) (Figure 4A, for summary see Supplementary Table S1). It is noteworthy to mention that plastid sections with extremely dense stroma were sometimes observed within the same cell along with plastid sections of moderate electron density. Plastids of leaf epidermis cells always had extremely electron-dense stroma with dark inclusions, clusters of plastoglobule-like ovale structures and peripheral vesicles or invaginations, and hardly distinguishable inner membranes (Figure 4B) which were sometimes stacked (to bithylakoids or rarely to low grana) (Supplementary Figure S1A). Plastids of the second leaf primordia of dark-forced shoots were etio-chloroplasts with electron-dense stroma, which contained grana that were occasionally interconnected with small, hardly visible prolamellar bodies (average diameter: 0.4 μm) (Supplementary Figure S1B). The fifth leaves of dark-forced shoots contained fully developed chloroplasts and no prolamellar body was observed in them (Supplementary Figure S1C). The presence of peripheral vesicles or invaginations was characteristic for dark-forced mesophyll plastids in all leaf layers (see Supplementary Figures S1A–C) but not for light-grown leaves of the same age (not shown). Phytoferritin inclusions were also present occasionally in the plastids of all studied leaf layers (Supplementary Figure S1D).

Capitate and peltate glandular hairs were observed already on primary leaves of the dark-forced shoots. Capitate glandular hairs had basically similar plastid structure to light-grown leaves (not shown), while some differences were observed in the plastids of fully differentiated peltate glandular hairs at the secretory stage as follows. The basal cells of the peltate glandular hairs of dark-forced leaves contained plastids with large electron-dense inclusions, and clusters of plastoglobule-like ovale structures (Figure 4C) and thus resembled plastids of basal cells in light-grown leaves (Figure 1G) and in dark-grown epidermis cells (Figure 4B). Plastid sections of the stalk cells resembled those present in light-grown plants (Figure 1H) but had in general very electron-densely stained stroma. The observed plastid sections sometimes contained only dark inclusions, but tubuloreticular membranes (diameter of approx. 0.9 μm) and clusters of plastoglobule-like ovale inclusions were also often present, as well as invaginations of the plastid envelope membrane (Figures 4D,E). Most plastid sections of the head cells of the peltate glandular hairs were similar to those observed in light-grown plants (Figure 1I) and had most typically extensive peripheral reticulum like structure, i.e., invaginations and vesicles at the plastid envelope (not shown). However, rarely prolamellar body-like dense membrane structures were also observed at the secretory phase (Figure 4F). Although we do not intend to provide an analysis of plastid development during glandular hair development, in this context we think it is important to mention that sometimes clusters of irregularly shaped, electron-dense vesicle like structures (average diameter: approx. 50 nm) were observed in peculiarly shaped, electron-dense plastids of the head cells at the late secretory phase (Figure 4G). This may be interesting as the enzymes involved in the biosynthesis of monoterpenes were immunolocalized to the cytoplasm of head cells and were absent from the stalk cells (Turner et al., 2012), however, the isoprenoid precursors needed for terpenoid biosynthesis may be produced by the plastids of head cells.

**FIGURE 5:**
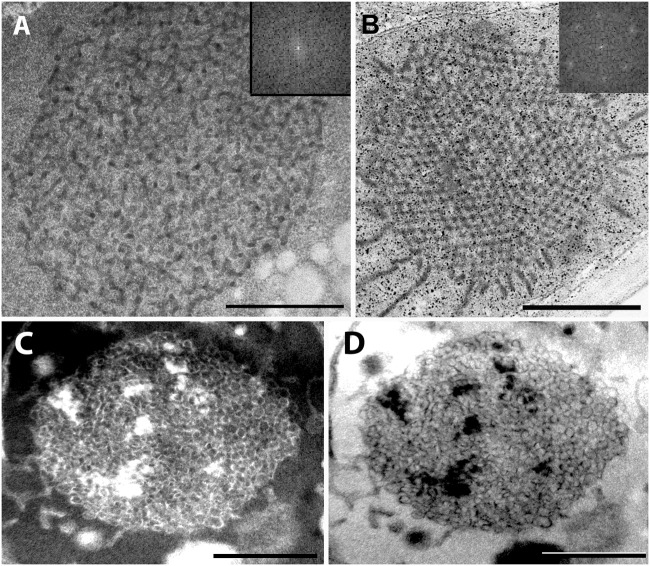
High magnification images of the tubuloreticular membrane structures in rosemary plastids. **(A)** Tubuloreticular inclusion in the stalk cell plastid of a peltate glandular hair of a light-grown leaf. **(B)** Prolamellar body from a mesophyll plastid of the cotyledon of 2-week-old rosemary seedling. **(C,D)** Tubuloreticular membrane structure in the stalk cell plastid of a peltate glandular hair of a dark-forced first leaf. **(D)** Represents the inverted (i.e., “negative”) image of **(C)**. Insets in **(A,B)** correspond to fast Fourier transformation (FFT) images of selected large central portions of the tubuloreticular membranes on pictures **(A,B)**, respectively. Scale bar: 0.5 μm.

For further comparison of the tubuloreticular structures in rosemary, we provide high magnification images of the prolamellar body-like membranes of the stalk cells of peltate glandular hairs of light-grown leaves (Figure 5A) and of dark-forced leaves (Figures 5C,D) as well as of “real” and highly regular prolamellar bodies (average diameter: 1.0 μm) of the etioplasts of fully etiolated cotyledons of 2-week-old seedlings (Figure 5B). The membranes seem to show inverse contrast [i.e., they have lightly staining membranes and electron-dense lumen – (Keresztes and Sárvári, 2001)], especially in dark-forced glandular hairs, therefore, in the latter case the “negative” image is also shown (compare Figures 5C,D). Unfortunately, the prolamellar bodies of mesophyll plastids of dark-forced primordia had low contrast when compared with the plastid stroma (Figure 4A), but in all cases the tubuloreticular arrangement of anastomosing membranes was evident. In order to characterize the regularity and periodicity of these membranes FFT of the image regions containing tubuloreticular structures was performed in ImageJ (see insets in Figures 5A,B). Periodic prolamellar body patterns appeared only in the FFT of prolamellar bodies of etio-chloroplasts of leaf mesophyll cells of dark-forced shoots (not shown) and in etioplasts of cotyledons of dark-germinated seedlings (Figure 5B, inset), no periodicity and regularity was observed by FFT in any tubuloreticular structure present in secretory tissues (e.g., Figure 5A, inset). The observed FFT image of the prolamellar bodies of cotyledons and etio-chloroplasts of dark-forced leaves resembled the structure observed in maize prolamellar bodies which clearly showed hexagonal symmetry (Schoefs et al., 2000).

Altogether, the tubuloreticular membranes of the etio-chloroplasts of dark-forced rosemary shoots had low contrast when compared to prolamellar bodies of etioplasts of several well characterized dark-grown model plants especially cereals (Gunning, 2001; Solymosi and Schoefs, 2008), while those in fully etiolated rosemary cotyledons clearly showed regular structure (Figure 5B). It is also clear that the tubuloreticular structures observed in glandular hairs (Figures 4D–F, 5A,C,D) have less regular and thus different structure from prolamellar bodies of mesophyll plastids of dark-forced leaves or cotyledons (Figures 4A, 5B). In addition, the tubular complexes present in stalk cells are reminiscent of the structure of prolamellar bodies being disrupted immediately after illumination (Gunning, 1965; Henningsen and Boynton, 1969) while neither their size or structure is influenced by dark-forcing. Similar loosely regular structures have been only rarely observed in secretory cells like resin-secreting cells of *Centrolobium* (Matos and Paiva, 2012). It should be noted, however, that the fixation, embedding and staining protocols developed for transmission electron microscopy may not be ideal for all kind of plant material, especially for those with electron-dense luminal substances (Keresztes and Sárvári, 2001; Solymosi et al., 2007) and that prolamellar body structure shows variation among species but also during development (Solymosi and Schoefs, 2008; Solymosi and Aronsson, 2013). Clearly, the observed tubuloreticular membranes of rosemary stalk cells and head cells are not closely resembling the clusters of electron-dense vesicles described as prolamellar body-like structures in the stalk cells of peppermint (Turner et al., 2000) or head cells (Figure 4G) or the more regular prolamellar body-like structures described in other secretory plastids (Hammond and Mahlberg, 1978; Vermeer and Peterson, 1979; Ascensao and Pais, 1982; Kim and Mahlberg, 1997; Solymosi and Köfalvi, 2017). To our knowledge this is the first report which compared systematically the different tubuloreticular membrane arrangements of secretory structures and etiolated tissues of the same plant and thus clearly pointed out similarities and differences in these structures. Furthermore, ImageJ and the application of FFT to transmission electron micrographs proved to be a good tool to distinguish between highly regular prolamellar bodies and more loosely arranged tubuloreticular membrane structures present in leucoplasts. Electron tomographic, 3D reconstruction of these tubuloreticular membranes under various illumination conditions could be used to further clarify their structure and interconnections with other plastid components (e.g., plastoglobuli).

Our data showed that plastid structure was influenced by dark-forcing in head cells of peltate glandular hairs and in the mesophyll cells of young leaves (compare Figures 1, 4). Instead of chloroplasts, etio-chloroplasts developed in mesophyll cells similarly to other dark-forced, fully or partially etiolated green tissues (Solymosi et al., 2006, 2007, 2012; Schoefs and Franck, 2008). More interestingly, tubuloreticular membranes occasionally appeared in the leucoplasts (secretory plastids) of the head cells of dark-forced peltate glandular hairs (Figure 4F). This latter proves that external stimuli such as light may influence plastid differentiation even in cells with obvious secretory function, and that in these structures proplastids may differentiate into etio-leucoplasts. This is not surprising if we consider the high developmental flexibility and plasticity of these organelles (Solymosi and Keresztes, 2012), however, to the best our knowledge such intermediary plastids or plastid transformation pathways have not been described earlier. Furthermore, the question can be raised whether the absence of light for 2 weeks also influenced the activity of these plastids, and the secretory process of the glandular hairs.

These data outline that in addition to experimental systems using cotyledons of etiolated seedlings (in case of which often there is not enough storage material for the first true leaves of dicot plants to emerge during prolonged dark growth), alternative systems like newly developed shoots of dark-forced adult plants may be also used. However, the growth of the shoot apical meristem may be inhibited by darkness (Mohammed et al., 2018). Also in case of rosemary it has to be outlined that dark-forcing of adult plants was only successful in certain periods of the year, probably at developmental stages when the shoots of the woody shrubs of rosemary also normally get elongated and develop new shoot parts (with new nodes and leaves).

### The Effect of Prolonged Darkness on the Essential Oil Composition of Rosemary Leaves

There are not many literature data on the effect of light on the essential oil composition of aromatic or medicinal plants, therefore, it was studied in dark-forced shoots and etiolated cotyledons and compared to data in light-grown shoots and cotyledons of the same age (Table 2). Light has a general influence on plant metabolism and plastid metabolites (Chen and Zhou, 2018) due to switch between autotrophic growth and heterotrophy, and 30 h dark treatment was shown to influence the phenolic diterpene content in rosemary (Loussouarn et al., 2017). However, these latter compounds are antioxidants and thus sensitive to light-induced oxidative stress (Loussouarn et al., 2017), while the biosynthesis and secretion of essential oil components (monoterpenes and sesquiterpenes) is a process that may proceed in slower time scale than 30 h and only in distinct developmental stages (McConkey et al., 2000). Due to the low quantity of biomass (e.g., small size and amount of dark-forced shoots and their small shoot tips and first leaf primordia) volatile analysis using the SPME technique proved to be adequate to evaluate and compare essential oil production of the different organs. It has to be noted, however, that plastids are important in producing the building blocs for isoprenoid biosynthesis and maybe even for the first steps of monoterpene biosynthesis, but further steps of monoterpene biosynthesis as well as the biosynthesis of sesquiterpenes is probably located to the cytoplasm (Turner et al., 2012; Lange and Turner, 2013; Sharifi-Rad et al., 2017; Lange and Srividya, 2019).

The main compounds of the essential oil of the light-grown fully developed (fifth) leaves were α-pinene (25.2%) and β-caryophyllene (13.1%) (Table 2). Further major components were 1.8-cineole, camphor and verbenone. Similar essential oil composition was reported earlier in rosemary leaves (Tucker and Maciarello, 1986; Boix et al., 2011; Affholder et al., 2013; Sagawa et al., 2013; Loussouarn et al., 2017), peltate glandular hairs (Sagawa et al., 2013) or even callus (Boix et al., 2013). However, essential oil composition strongly varies among cultivars (Tucker and Maciarello, 1986) and developmental stages or even during seasons (Lakusic and Slavkovska, 2013). Dark-forcing did not influence significantly the essential oil composition of these fully developed leaves (Table 2).

On the other hand, the essential oil of the young leaves (i.e., shoot apex and first leaf primordia) contained sesquiterpenes, mainly β-caryophyllene in a significantly higher ratio (22.1–22.3%) compaired to older leaves. There was no significant difference in the ratio of monoterpenes and sesquiterpenes between the light-grown and dark-forced young leaves (Table 2). The main component of the dark-forced young leaves was bornyl acetate (26.1%), which was present in slightly but not significantly lower ratio in the essential oils of light-grown younger leaves, and in significantly lower amounts in light-grown or dark-forced older leaves (2.5% and 2.1%, respectively). The pharmacologically important compounds such as α-pinene, 1.8-cineole and camphore, which are responsible for the antirheumatic effect of rosemary leaf (Borges et al., 2018; De Oliveira et al., 2019), were present in a low ratio in the dark-forced leaves, especially in the youngest leaves. Therefore, from a therapeutic aspect, the lack of light adversely affects the essential oil composition of rosemary leaves. Similarly, young leaves at the shoot apex had different essential oil composition from fully developed leaves. This latter is not surprising as the development of glandular hairs, the biosynthesis and secretion of essential oils is a time-consuming procedure and thus the essential oil composition has been shown to depend on the age of the studied material in several different Lamiaceae species [mint – (Gershenzon et al., 2000; McConkey et al., 2000); basil – (Werker et al., 1993)].

The cotyledons of light-germinated and dark-germinated seedlings had similar but somewhat special essential oil composition with bornyl acetate, β-pinene and δ-cadinene as major compounds (Table 2). Several compounds present in leaves of different age (e.g., myrcene, borneol, izopinocamphon, and verbenone) were not detected in cotyledons, while other minor sesquiterpene compounds (e.g., aristolene, ylangene, β- and γ-muurolene, δ-cadinene, and τ-cadinol) were only detected in cotyledons but not in leaves (Table 2). This outlines that in addition to their extremely small size, cotyledons are also not suitable sources for conventional therapeutic essential oils from rosemary as they have completely different essential oil composition. High bornyl acetate contents were observed in peltate glandular hairs of leaves and stipules of rosemary in a work in which hot water extraction and SPME-GC/MS was used for quantification (Sagawa et al., 2013). Bornyl acetate is a highly added value compound used as food additive, flavoring agent and also in the perfume and cosmetic industries due to its pine scent. Recent data show that it may also have some therapeutic interest (Karan et al., 2018). For several compounds an ascending (or in other cases descending) relative contribution was observed as follows: dark-germinated cotyledons = light-germinated cotyledons < dark-forced young shoots < light-grown young shoots < fully developed leaves (both on light-grown and dark-forced shoots).

Our data thus outine that not only the age of the studied tissue, its type (i.e., leaves vs. cotyledons) but also dark-forcing or dark-germination of rosemary influence the biosynthesis of the essential oil compounds (Table 2) and thus its potential therapeutic, culinary or aromatic uses.

## Conclusion

The data of this manuscript provide insights into the light-regulation of plastid differentiation as well as plant metabolism (chlorophyll biosynthesis in different organs and essential oil production by secretory structures). These processes were systematically studied and compared at two distinct developmental stages, i.e., in cotyledons of germinating seeds which had limited lifetime and storage materials, and in shoot apical meristems and “real” leaves of adult dicot plants. Therefore, comparative data in different organs (cotyledons and leaves) and in addition at different developmental stages of the latter (young, differentiating leaf primordia and fully developed leaves) were also provided.

(1) The “real” etioplasts of the mesophyll cells of etiolated rosemary cotyledons had highly regular prolamellar bodies and accumulated high amounts of protochlorophyll/ide and were fully devoid of chlorophylls, while (2) the proplastids of the previously illuminated (i.e., light-grown) shoot tip of adult dark-forced plants only differentiated into etio-chloroplasts. The latter accumulated low amounts of protochlorophyll/ide, contained only residual chlorophyllous pigments (that were transferred to the mesophyll cell plastids of the developing leaf primordia by plastid division from the shoot apical meristem), and as a consequence bithylakoids and grana interconnected with less regular and smaller prolamellar bodies. This shows clear differences in the two distinct etiolation systems in spite of some (morphological) similarities (elongation of internodes, pale color of dark-grown tissues, appearance of prolamellar bodies and accumulation of photoactive protochlorophyllide). This is probably due to differences in several other factors including carotenoid content, specific regulatory and metabolic pathways of the different organs which may be subject to further studies. It has to be noted, however, that (3) the structure of the chloroplasts of fully developed green leaves (fifth leaves) close to their final chlorophyll contents and with stable differentiation and activity of their photosynthetic apparatus was not influenced by 2 weeks of dark forcing, indicating a developmental milestone in chloroplast differentiation after which the appearance of prolamellar bodies cannot be induced (Ikeda, 1970, 1971).

(4) On the other hand, we provided the first detailed ultrastructural description of secretory plastids of rosemary and (5) the first evidence for the presence of tubuloreticular membrane complexes in the stalk cells of peltate glandular hairs of rosemary leaves under natural light conditions. (6) Furthermore, we proved that the absence of light did not strongly influence the structure of these tubular complexes, indicating that in contrast to mesophyll cells, plastid differentiation in the stalk cells was not influenced by darkness. (7) In addition, these tubular complexes of stalk cell plastids were clearly different and much less regular than the prolamellar bodies observed in etiolated or dark-forced mesophyll cells. (8) Rarely tubuloreticular membranes were observed in the head cells of the peltate glandular hairs during dark-forcing, indicating that in these cells light indeed influenced plastid structure at least at some stages of glandular hair development.

It is not yet clear what induces the formation of the tubuloreticular membranes, i.e., tubular complexes in the stalk cells of the peltate glandular hairs of rosemary or mint (Amelunxen, 1965; Turner et al., 2000, 2012). The short chain reductases required for the biosynthesis of monoterpenes were localized in the cytoplasm of head cells and not in the stalk cells (Turner et al., 2012), therefore, it is probable that these tubular complexes are not directly related to the biosynthesis of monoterpene compounds of the essential oils. Stalk cells are involved in the production of suberin and cutin-like hydrophobic substances that serve as apoplastic barrier to the essential oils (Serrato-Valenti et al., 1997; Bisio et al., 1999) and require intensive *de novo* fatty acid synthesis of plastids (Vishwanath et al., 2015; Fich et al., 2016), processes that may result in alterations of membrane composition or structure similarly to etioplasts in which also intensive biosynthesis and storage of membrane components occurs (Solymosi and Schoefs, 2010; Solymosi and Aronsson, 2013). However, further investigations are required to determine the factors inducing the formation of the tubular complexes in stalk cells.

Finally, (9) we identified cotyledons and dark-forced young leaves as potential sources of some industrially important compounds (bornyl acetate) which are present in much lower proportion in the leaves of light-grown plants. Our data indicated that (10) light, developmental stage (i.e., young leaf primordia or fully developed leaves) and organ type (i.e., leaves vs. cotyledons) all influence the essential oil composition of rosemary. Based on these observations we can conclude that young leaves and cotyledons as well as dark-forced or low-light grown plants may have different essential oil profile from normal light-grown plants. This may be considered when rosemary is grown under low light conditions (shaded areas or under laboratory conditions or glasshouses) or is moved indoor during winter in colder climates, and also outlines the general importance of light and developmental stage in plant secondary metabolism.

## Data Availability Statement

The datasets generated for this study are available on request to the corresponding author.

## Author Contributions

KS conceived the study, designed the experiments, and did transmission electron microscopy. AB carried out and evaluated analyses related to the essential oil composition. AD, MP, and KS performed the spectroscopic measurements and analyses. AS carried out photosynthetic activity measurements. AB and KS wrote the manuscript which was later edited and approved by all authors.

## Conflict of Interest

The authors declare that the research was conducted in the absence of any commercial or financial relationships that could be construed as a potential conflict of interest.

## References

[B1] Affholder M.-C. Prudent P. Masotti V. Coulomb B. Rabier J. (2013). Transfer of metals and metalloids from soil to shoots in wild rosemary (*Rosmarinus officinalis* L.) growing on a former lead smelter site: human exposure risk. *Sci. Total Environ.* 454–455 219–229. 10.1016/j.scitotenv.2013.02.086 23542674

[B2] Almsherqi Z. A. Kohlwein S. D. Deng Y. (2006). Cubic membranes: a legend beyond the flatland of cell membrane organization. *J. Cell Biol.* 173 839–844. 10.1083/jcb.200603055 16785319 PMC2063909

[B3] Almsherqi Z. A. Landh T. Kohlwein S. D. (2009). Cubic membranes: the missing dimension of cell membrane organization. *Int. Rev. Cell Mol. Biol.* 274 275–342. 10.1016/S1937-6448(08)02006-6 19349040 PMC7105030

[B4] Amelunxen F. (1965). Elektronenmikroskopische Untersuchungen an den Drüsenshuppen von *Mentha piperita* L. *Planta Med.* 13 457–473.

[B5] Ascensao L. Marques N. Pais M. S. (1997). Peltate glandular trichomes of Leonotis leonurus leaves: ultrastructure and histochemical characterization of secretions. *Int. J. Plant Sci.* 158 249–258. 10.1086/297436

[B6] Ascensao L. Pais M. S. (1998). The leaf capitate trichomes of Leonotis leonurus: histochemistry, ultrastructure and secretion. *Ann. Bot.* 81 263–271. 10.1006/anbo.1997.0550

[B7] Ascensao L. Pais M. S. S. (1982). Secretory trichomes from *Artemisia crithmifolia*: some ultrastructural aspects. *Bull. Soc. Bot. France* 129 83–87. 10.1080/01811789.1982.10826552

[B8] Bisio A. Corallo A. Gastaldo P. Romussi G. Ciarallo G. Fontana N. (1999). Glandular hairs and secreted material in *Salvia blepharophylla* Brandegee ex Epling grown in Italy. *Ann. Bot.* 83 441–452. 10.1006/anbo.1998.0838

[B9] Björkman O. Demmig B. (1987). Photon yield of O2 evolution and chlorophyll fluorescence characteristics at 77 K among vascular plants of diverse origins. *Planta* 170 489–504. 10.1007/BF00402983 24233012

[B10] Böddi B. Lindsten A. Ryberg M. Sundqvist C. (1989). On the aggregational states of protochlorophyllide and its protein complexes in wheat etioplasts. *Physiol. Plant.* 76 135–143. 10.1111/j.1399-3054.1989.tb05622.x

[B11] Böddi B. Lindsten A. Sundqvist C. (1999). Chlorophylls in dark-grown epicotyl and stipula of pea. *J. Photochem. Photobiol. B Biol.* 48 11–16. 10.1016/s1011-1344(99)00002-0

[B12] Boix Y. F. Arruda R. C. O. Defaveri A. C. A. Sato A. Lage C. L. S. Victório C. P. (2013). Callus in *Rosmarinus officinalis* L. (*Lamiaceae*): a morphoanatomical. histochemical and volatile analysis. *Plant Biosyst.* 147 751–757. 10.1080/11263504.2012.751067

[B13] Boix Y. F. Carina A. Defaveri A. (2011). Glandular trichomes of *Rosmarinus officinalis* L.: anatomical and phytochemical analyses of leaf volatiles. *Plant Biosyst.* 145 848–856. 10.1080/11263504.2011.584075

[B14] Boor P. J. Ferrans V. J. Jones M. Kawanami O. Thiedemann K.-U. Herman E. H. (2018). Tubuloreticular structures in myocardium: an ultrastructural study. *J. Mol. Cell Cardiol.* 11 967–976. 10.1016/0022-2828(79)90388-2 522138

[B15] Borges R. S. Lorena B. Ortiz S. César A. Pereira M. Keita H. (2018). Rosmarinus officinalis essential oil: a review of its phytochemistry, anti-inflammatory activity, and mechanisms of action involved. *J. Ethnopharmacol.* 229 29–45. 10.1016/j.jep.2018.09.038 30287195

[B16] Briantais J.-M. Vernotte C. Krause G. Weis E. (1986). “Chlorophyll a fluorescence of higher plants: chloroplasts and leaves,” in *Light Emission by Plants and Bacteria*, eds Govindjee J. Fork A. D., (Cambridge, MA: Academic Press), 539–583. 10.1016/b978-0-12-294310-2.50024-x

[B17] Brouers M. Michel-Wolwertz M.-R. (1983). Estimation of protochlorophyll(ide) contents in plant extracts; re-evaluation of the molar absorption coefficient of protochlorophyll(ide). *Photosynth. Res.* 4 265–270. 10.1007/bf00052130 24458496

[B18] Chen Y. Zhou B. (2018). Formation and change of chloroplast-located plant metabolites in response to light conditions. *Int. J. Mol. Sci.* 19:654. 10.3390/ijms19030654 29495387 PMC5877515

[B19] Chong K. Almsherqi Z. A. Shen H. Deng Y. (2017). Cubic membrane formation supports cell survival of amoeba Chaos under starvation-induced stress. *Protoplasma* 255 517–525. 10.1007/s00709-017-1169-x 28914376

[B20] De Oliveira J. R. Esteves S. Camargo A. (2019). *Rosmarinus officinalis* L. (rosemary) as therapeutic and prophylactic agent. *J. Biomed. Sci.* 26:5. 10.1186/s12929-019-0499-8 30621719 PMC6325740

[B21] Deng Y. Almsherqi Z. A. Ng M. M. L. Kohlwein S. D. (2010). Do viruses subvert cholesterol homeostasis to induce host cubic membranes? *Trends Cell Biol.* 20 371–379. 10.1016/j.tcb.2010.04.001 20434915 PMC7127466

[B22] Dumbravǎ D. Moldovan C. (2012). Vitamin C, chlorophylls, carotenoids and xanthophylls content in some basil (*Ocimum basilicum* L) and rosemary (*Rosmarinus officinalis* L) leaves extracts. *J. Agroalim. Process. Technol.* 18 253–258.

[B23] Eady R. A. J. Odland G. F. (1975). Intraendothelial tubular aggregates in experimental wounds. *Br. J. Dermatol.* 93 165–173. 10.1111/j.1365-2133.1975.tb06736.x 169873

[B24] Ferroni L. Baldisserotto C. Pantaleoni L. Fasulo M. P. Fagioli P. Pancaldi S. (2009). Degreening of the unicellular alga Euglena gracilis: thylakoid composition, room temperature fluorescence spectra and chloroplast morphology. *Plant Biol.* 11 631–641. 10.1111/j.1438-8677.2008.00152.x 19538401

[B25] Fich E. A. Segerson N. A. Rose J. K. C. (2016). The plant polyester cutin: biosynthesis, structure, and biological roles. *Annu. Rev. Plant Biol.* 67 207–233. 10.1146/annurev-arplant-043015-111929 26865339

[B26] Gershenzon J. McConkey M. E. Croteau R. B. (2000). Regulation of monoterpene accumulation in leaves of peppermint. *Plant Physiol.* 122 205–213. 10631264 10.1104/pp.122.1.205 PMC58859

[B27] Giuliani C. Pellegrino R. Tirillini B. Maleci L. (2008). Micromorphological and chemical characterisation of *Stachys recta* L. subsp. serpentini (Fiori) Arrigoni in comparison to *Stachys recta* L. subsp. recta (*Lamiaceae*). *Flora* 203 376–385. 10.1016/j.flora.2007.07.001

[B28] Gunning B. (1965). The greening process in plastids. 1. The structure of the prolamellar body. *Protoplasma* 60 111–130. 10.1007/bf01248133

[B29] Gunning B. (2001). Membrane geometry of “open” prolamellar bodies. *Protoplasma* 215 4–15. 10.1007/bf01280299 11732064

[B30] Hammond C. T. Mahlberg P. G. (1978). Ultrastructural development of capitate glandular hairs of *Cannabis sativa* L. (*Cannabaceae*). *Amer. J. Bot.* 65 140–151. 10.1002/j.1537-2197.1978.tb06051.x

[B31] Henningsen K. Boynton J. (1969). Macromolecular physiology of plastids VII. The effect of brief illumination on plastids of dark-grown barley leaves. *J. Cell Sci.* 5 757–793. 5361241 10.1242/jcs.5.3.757

[B32] Huang S.-S. Kirchoff B. Liao J.-P. (2008). The capitate and peltate glandular trichomes of *Lavandula pinnata* L. (*Lamiaceae*): histochemistry. ultrastructure and secretion. *J. Torrey Bot. Soc.* 135 155–167. 10.3159/07-ra-045.1

[B33] Ikeda T. (1970). Changes in fine structure of prolamellar body in relation to the formation of the chloroplast. *Bot. Mag. Tokyo* 83 1–9. 10.15281/jplantres1887.83.1

[B34] Ikeda T. (1971). Prolamellar body formation under different light and temperature conditions. *Bot. Mag. Tokyo* 84 363–376. 10.15281/jplantres1887.84.363 13756693

[B35] Kakuszi A. Solymosi K. Böddi B. (2017). Transformation of plastids in soil-shaded lowermost hypocotyl segments of bean (*Phaseolus vulgaris*) during a 60-day cultivation period. *Physiol. Plant.* 159 483–491. 10.1111/ppl.12519 27734513

[B36] Karan T. Yildiz I. Aydin A. (2018). Inhibition of various cancer cells proliferation of bornyl acetate and essential oil from Inula graveolens (*Linnaeus*) Desf. *Rec. Nat. Prod.* 3 273–283. 10.25135/rnp.30.17.09.057

[B37] Kashina T. K. Danilova M. F. (1993). Ultrastructure of glandular hair plastids and nictophylness of *Perilla ocymoides* L. *Russ. J. Plant Physiol.* 40 785–790.

[B38] Keresztes Á Sárvári É (2001). Investigations into the»inverse contrast«of chloroplast thylakoids. *Acta Bot. Croat.* 60 253–265.

[B39] Kim E.-S. Mahlberg P. G. (1997). Plastid development in disc cells of glandular trichomes of Cannabis (*Cannabaceae*). *Mol. Cells* 7 352–359. 9264022

[B40] Kolalite M. (1998). Comparative analysis of ultrastructure of glandular trichomes in two Nepeta cataria chemotypes (*N. cataria* and *N. catena var. citriodora*). *Nord. J. Bot.* 18 589–598. 10.1111/j.1756-1051.1998.tb01542.x

[B41] Kovács E. Keresztes A. (2002). Effect of gamma and UV-B/C radiation on plant cells. *Micron* 33 199–210. 10.1016/s0968-4328(01)00012-9 11567888

[B42] Kowalewska Ł. Bykowski M. Mostowska A. (2019). Spatial organization of thylakoid network in higher plants. *Bot. Lett.* 166 326–343. 10.1080/23818107.2019.1619195 29982996

[B43] Lakusic D. Slavkovska V. (2013). Seasonal variations in the composition of the essential oils of rosemary (*Rosmarinus officinalis*, Lamiaceae). *Nat. Prod. Commun.* 8 131–134. 23472478

[B44] Lange B. M. Srividya N. (2019). Enzymology of monoterpene functionalization in glandular trichomes. *J. Exp. Bot.* 70 1095–1108. 10.1093/jxb/ery436 30624688

[B45] Lange B. M. Turner G. W. (2013). Terpenoid biosynthesis in trichomes-current status and future opportunities. *Plant Biotechnol. J.* 11 2–22. 10.1111/j.1467-7652.2012.00737.x 22979959

[B46] Larsson M. Larsson K. (2014). Periodic minimal surface organizations of the lipid bilayer at the lung surface and in cubic cytomembrane assemblies. *Adv. Colloid Interface Sci.* 205 68–73. 10.1016/j.cis.2013.07.003 23910375

[B47] Lindquist E. Solymosi K. Aronsson H. (2016). Vesicles are persistent features of different plastids. *Traffic* 17 1125–1138. 10.1111/tra.12427 27405297

[B48] Loussouarn M. Krieger-Liszkay A. Svilar L. Bily A. Birti S. Havaux M. (2017). Carnosic acid and carnosol, two major antioxidants of rosemary, act through different mechanisms. *Plant Physiol.* 175 1381–1394. 10.1104/pp.17.01183 28916593 PMC5664485

[B49] Luu J. Bockus D. Remington F. Bean M. Hammar S. P. (1989). Tubuloreticular structures and cylindrical confronting cisternae: a review. *Prog. Pathol.* 20 617–627. 10.1016/0046-8177(89)90148-2 2661406

[B50] Marin M. Koko V. Duleti S. Marin P. D. Ran D. Dajic-Stevanovic Z. (2006). Glandular trichomes on the leaves of *Rosmarinus officinalis*: morphology, stereology and histochemistry. *South Afr. J. Bot.* 72 378–382. 10.1016/j.sajb.2005.10.009

[B51] Matos E. Paiva E. (2012). Structure, function and secretory products of the peltate glands of *Centrolobium tomentosum* (*Fabaceae. Faboideae*). *Austr. J. Bot.* 60 301–309.

[B52] McConkey M. E. Gershenzon J. Croteau R. B. (2000). Developmental regulation of monoterpene biosynthesis in the glandular trichomes of peppermint. *Plant Physiol.* 122 215–224. 10.1104/pp.122.1.215 10631265 PMC58860

[B53] Mohammed B. Bilooei S. F. Dóczi R. Grove E. Railo S. Palme K. (2018). Converging light, energy and hormonal signaling control meristem activity, leaf initiation, and growth. *Plant Physiol.* 176 1365–1381. 10.1104/pp.17.01730 29284741 PMC5813583

[B54] Park H. Kreunen S. S. Cuttriss A. J. Dellapenna D. Pogson B. J. (2002). Identification of the carotenoid isomerase provides insight into carotenoid biosynthesis, prolamellar body formation, and photomorphogenesis. *Plant Cell* 14 321–332. 10.1105/tpc.010302 11884677 PMC152915

[B55] Porra R. Thompson W. Kriedemann P. (1989). Determination of accurate extinction coefficients and simultaneous equations for assaying chlorophylls *a* and *b* extracted with four different solvents: verification of the concentration of chlorophyll standards by atomic absorption spectroscopy. *Biochim. Biophys. Acta Bioenergetics* 975 384–394. 10.1016/s0005-2728(89)80347-0

[B56] Sagawa T. Ikeda H. Hiraoka T. Hayakawa K. (2013). Study of rosemary peltate glandular trichomes using combined morphological and chemical approach. *Food Sci. Technol. Res.* 19 491–495. 10.3136/fstr.19.491

[B57] Schnepf E. (1961). Plastidenstrukturen bei Passiflora. *Protoplasma* 54 310–313.

[B58] Schoefs B. Franck F. (2008). The photoenzymatic cycle of NADPH:protochlorophyllide oxidoreductase in primary bean leaves (*Phaseolus vulgaris*) during the first days of photoperiodic growth. *Photosynth. Res.* 96 15–26. 10.1007/s11120-007-9274-x 17978860

[B59] Schoefs B. Hucek S. Husak M. (2000). “Determination of unit cell size of prolamellar bodies from maize leaves,” in *EUREM 12*, (Brno), B137–B138.

[B60] Scrutton N. S. Groot M. L. Heyes D. J. (2012). Excited state dynamics and catalytic mechanism of the light-driven enzyme protochlorophyllide oxidoreductase. *Phys. Chem. Chem. Phys.* 14 8818–8824. 22419074 10.1039/c2cp23789j

[B61] Serrato-Valenti G. Bisio A. Cornara L. Ciarallo G. (1997). Structural and histochemical investigation of the glandular trichomes of *Salvia aurea* L. leaves. and chemical analysis of the essential oil. *Ann. Bot.* 79 329–336. 10.1006/anbo.1996.0348

[B62] Sharifi-Rad J. Sureda A. Tenore G. C. Daglia M. Sharifi-Rad M. Valussi M. (2017). Biological activities of essential oils: from plant chemoecology to traditional healing systems. *Molecules* 22:E70. 10.3390/molecules22010070 28045446 PMC6155610

[B63] Solymosi K. Aronsson H. (2013). “Etioplasts and their significance in chloroplast biogenesis,” in *Plastid Development in Leaves During Growth and Senescence. Advances in Photosynthesis and Respiration*, Vol. 36 eds Biswal B. Krupinska K. Biswal U. C., (Dordrecht: Springer), 39–71. 10.1007/978-94-007-5724-0_3

[B64] Solymosi K. Böddi B. (2006). Optical properties of bud scales and protochlorophyll(ide) forms in leaf primordia of closed and opened buds. *Tree Physiol.* 26 1075–1085. 10.1093/treephys/26.8.1075 16651257

[B65] Solymosi K. Bóka K. Böddi B. (2006). Transient etiolation: protochlorophyll(ide) and chlorophyll forms in differentiating plastids of closed and breaking leaf buds of horse chestnut (*Aesculus hippocastanum*). *Tree Physiol.* 26 1087–1096. 10.1093/treephys/26.8.1087 16651258

[B66] Solymosi K. Keresztes Á (2012). Plastid structure, diversification and interconversions II. Land plants. *Curr. Chem. Biol.* 6 187–204. 10.2174/2212796811206030003

[B67] Solymosi K. Köfalvi A. (2017). Cannabis: a treasure trove or Pandora’s box? *Mini Rev. Med. Chem.* 17 1223–1291. 10.2174/1389557516666161004162133 27719666

[B68] Solymosi K. Martinez K. Kristóf Z. Sundqvist C. Böddi B. (2004). Plastid differentiation and chlorophyll biosynthesis in different leaf layers of white cabbage (*Brassica oleracea* cv. *capitata*). *Physiol. Plant.* 121 520–529. 10.1111/j.0031-9317.2004.00349.x

[B69] Solymosi K. Morandi D. Bóka K. Böddi B. Schoefs B. (2012). High biological variability of plastids, photosynthetic pigments and pigment forms of leaf primordia in buds. *Planta* 235 1035–1049. 10.1007/s00425-011-1559-9 22160501

[B70] Solymosi K. Schoefs B. (2008). “Prolamellar body: a unique plastid compartment, which does not only occur in dark- grown leaves,” in *Plant Cell Compartments - Selected Topics*, ed. Schoefs B., (Trivandrum: Research Signpost), 151–202.

[B71] Solymosi K. Schoefs B. (2010). Etioplast and etio-chloroplast formation under natural conditions: The dark side of chlorophyll biosynthesis in angiosperms. *Photosynth. Res.* 105 143–166. 10.1007/s11120-010-9568-2 20582474

[B72] Solymosi K. Vitányi B. Hideg É. Böddi B. (2007). Etiolation symptoms in sunflower (Helianthus annuus) cotyledons partially covered by the pericarp of the achene. *Ann. Bot.* 99 857–867. 10.1093/aob/mcm034 17452377 PMC2802920

[B73] Stpiczynska M. Milanesi C. Faleri C. Cresti M. (2005). Ultrastructure of the nectary spur of Platanthera chlorantha (Custer) Rchb. (*Orchidaceae*) during successive stages of nectar secretion. *Acta Biol. Crac. Ser. Bot.* 47 111–119.

[B74] Tucker A. Maciarello M. J. (1986). The essential oils of some rosemary cultivars. *Flavour Fragr. J.* 1 137–142. 10.1002/ffj.2730010402 24584866

[B75] Turner G. W. Davis E. M. Croteau R. B. (2012). Immunocytochemical localization of short-chain family reductases involved in menthol biosynthesis in peppermint. *Planta* 235 1185–1195. 10.1007/s00425-011-1567-9 22170164

[B76] Turner G. W. Gershenzon J. Croteau R. B. (2000). Development of peltate glandular trichomes of peppermint. *Plant Physiol.* 124 665–680. 10.1104/pp.124.2.665 11027716 PMC59172

[B77] Vermeer J. Peterson R. L. (1979). Glandular trichomes on the inflorescence of Chrysanthemum morifolium cv dramatic (Compositae). 1. *Dev. Morphol. Can. J. Bot.* 57 705–713. 10.1139/b79-090

[B78] Vishwanath S. J. Delude C. Rowland O. (2015). Suberin: biosynthesis, regulation, and polymer assembly of a protective extracellular barrier. *Plant Cell Rep.* 34 573–586. 10.1007/s00299-014-1727-z 25504271

[B79] Werker E. Putievsky E. Ravid U. Dudai N. Katzir I. (1993). Glandular hairs and essential oil in developing leaves of *Ocimum basilicum* L. (Lamiaceae). *Annals of Botany.* 71 43–50. 10.1006/anbo.1993.1005

[B80] Werker E. Ravid U. Putievsky E. (1985). Structure of glandular hairs and identification of the main components of their secreted material in some species of the Labiatae family. *Isr. J. Bot.* 34 31–45.

