# Supplementary material for: The Effect of Light on Plastid Differentiation, Chlorophyll Biosynthesis, and Essential Oil Composition in Rosemary (Rosmarinus officinalis) Leaves and Cotyledons
Source: Front Plant Sci. 2020 Mar 3;11:196. doi: 10.3389/fpls.2020.00196 (PMC7063033; doi:10.3389/fpls.2020.00196)
Supplement: Supplementary file 1 [file Data_Sheet_1.pdf]

**Supplementary Material**

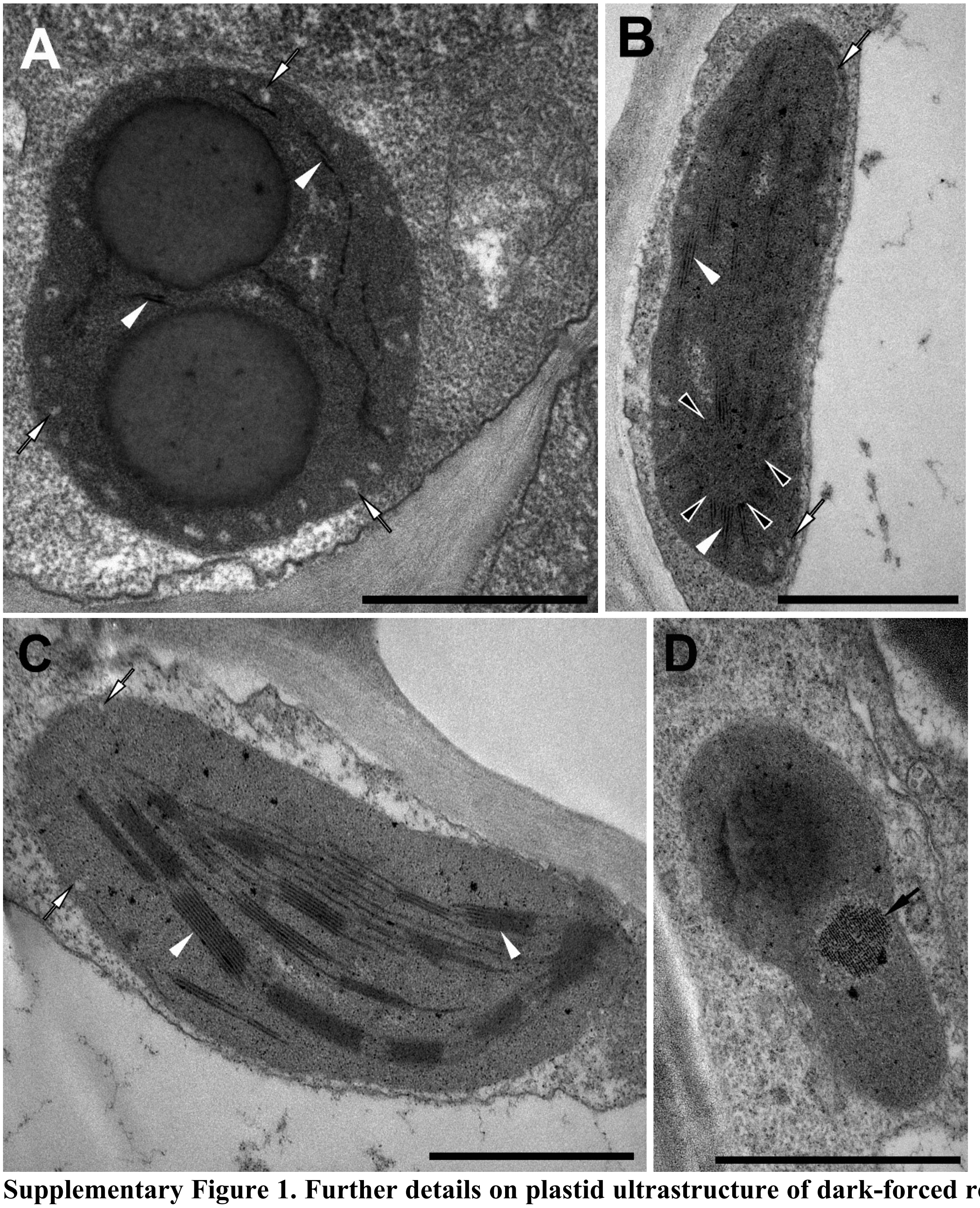


**Supplementary Figure 1. Further details on plastid ultrastructure of dark-forced rosemary (*Rosmarinus officinalis*) shoots developed on adult plants during 2 weeks of growth in complete darkness. (A)** Plastid from the adaxial epidermis of first leaf primordium of a dark-forced shoot. **(B)** Etio-chloroplast from the mesophyll cell of a second leaf of a dark-forced shoot. **(C)** Chloroplast from the mesophyll cell of the fifth leaf of a dark-forced shoot. **(D)** Plastid from a phloem parenchyma cell of a second leaf of a dark-forced shoot. White arrowhead: bithylakoids or grana; white arrow: peripheral vesicles or invaginations, black arrowhead: small, hardly distinguishable prolamellar body; black arrow: phytoferritin. Scale bar: 1 μm.

**Supplementary Table 1. Summarizing the observations on plastid types and ultrastructural features in different cells of rosemary (*Rosmarinus officinalis*) leaves under various conditions.**

| | Leaf mesophyll cell | Epidermis cell | Capitate glandular hair | | | Peltate glandular hair | | |
|---|---|---|---|---|---|---|---|---|
| | | | Basal cell | Stalk cell | Head Cell | Basal cell | Stalk cell | Head cell |
| **Light-grown samples** | | | | | | | | |
| **Young and old leaves of adult plants** | Chloroplast | Chloroplast (electron-dense stroma, thylakoids and grana with inverse contrast, invaginations) | Like in epidermis | Leucoplast (few single thylakoid-like structures, electron-dense inclusion) | | Like in epidermis (with low grana and electron-dense inclusion) | Leucoplast (tubulo-reticular membranes, electron dense inclusion) | Leucoplast (electron-dense stroma, invaginations) |
| **Cotyledons of seedlings** | Chloroplast | Not analyzed | | | | | | |
| **Dark-forced or dark-germinated samples** | | | | | | | | |
| **First leaf and shoot tip of dark-forced shoot** | Etio-chloroplast (electron-dense stroma, prolamellar body with /bi/thylakoids, invaginations) | Like in light (with invaginations, electron-dense stroma, lower grana) | Like in light | | | Like in light and in epidermis cells | Like in light (more electron-dense stroma, invaginations) | Like in light (rarely prolamellar body-like structures appeared) |
| **Second leaf of dark-forced shoot** | Etio-chloroplast (small prolamellar body with grana, invaginations) | Not analyzed | | | | | | |
| **Fifth leaf of dark-forced shoot** | Chloroplast (invaginations) | Not analyzed | | | | | | |
| **Cotyledon of etiolated seedling** | Etioplast (regular prolamellar body) | Not analyzed | | | | | | |
